# High-Sensitivity Troponin 1 as a Potential Prognostic Biomarker for HFpEF in Low-Income Rural Settings

**DOI:** 10.1016/j.jaccas.2025.105605

**Published:** 2025-10-07

**Authors:** Arjun Mallasandra Balakrishna, Sreekanth B. Shetty, Deepak Krishnamurthy

**Affiliations:** a Department of Medicine, Akash Institute of Medical Sciences, Devanahalli, India; b Cardiology, Sakra World Hospital, Bangalore, India; c Cardiology, KIMS Hospital, Bangalore, India

**Keywords:** acute heart failure, chronic heart failure, diastolic heart failure

## Abstract

**Background:**

Heart failure causes high morbidity and mortality globally, especially in low- and middle-income countries, highlighting the need for affordable, accessible, and effective biomarkers.

**Objectives:**

To evaluate the prognostic utility of high-sensitivity cardiac troponin 1 (hs-cTn1) levels in predicting rehospitalization, major adverse cardiovascular event (MACE), and mortality rates among patients who have heart failure with preserved ejection fraction (HFpEF).

**Methods:**

In a prospective observational study, 94 patients diagnosed with HFpEF were stratified based on their admission hs-cTn1 level into 4 groups. The patients were evaluated for 12 months to study rehospitalization rates, incidence of MACE, and mortality.

**Results:**

Patients with hs-cTn1 level >30 ng/L exhibited the highest rates of rehospitalization (72.7%), MACE (81.8%), and mortality (36.4%). A stepwise decrease in these events was observed with lower hs-cTn1 levels. Statistical analysis confirmed significant associations between hs-cTn1 concentrations and all measured outcomes.

**Conclusions:**

Results indicated that hs-cTn1 serves as a potent prognostic biomarker for HFpEF, with higher levels indicating greater risks of rehospitalization, MACE, and mortality.

**Take-Home Message:**

Elevated high-sensitivity cardiac troponin 1 (hs-cTn1) levels are strongly associated with increased risks of rehospitalization, major adverse cardiovascular events, and mortality in heart failure patients. Given its affordability and accessibility, hs-cTn1 testing can emerge as a practical tool for early risk stratification in resource-limited settings.

## Graphical Abstract

**Figure:**
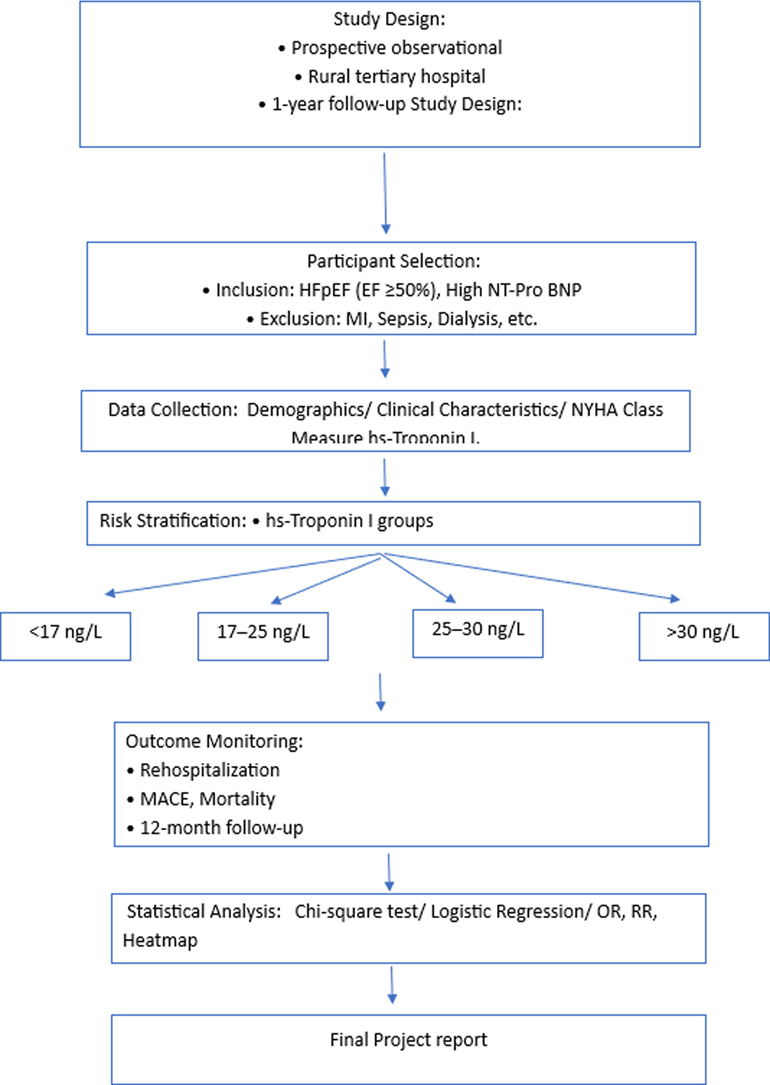

**Visual Summary:**
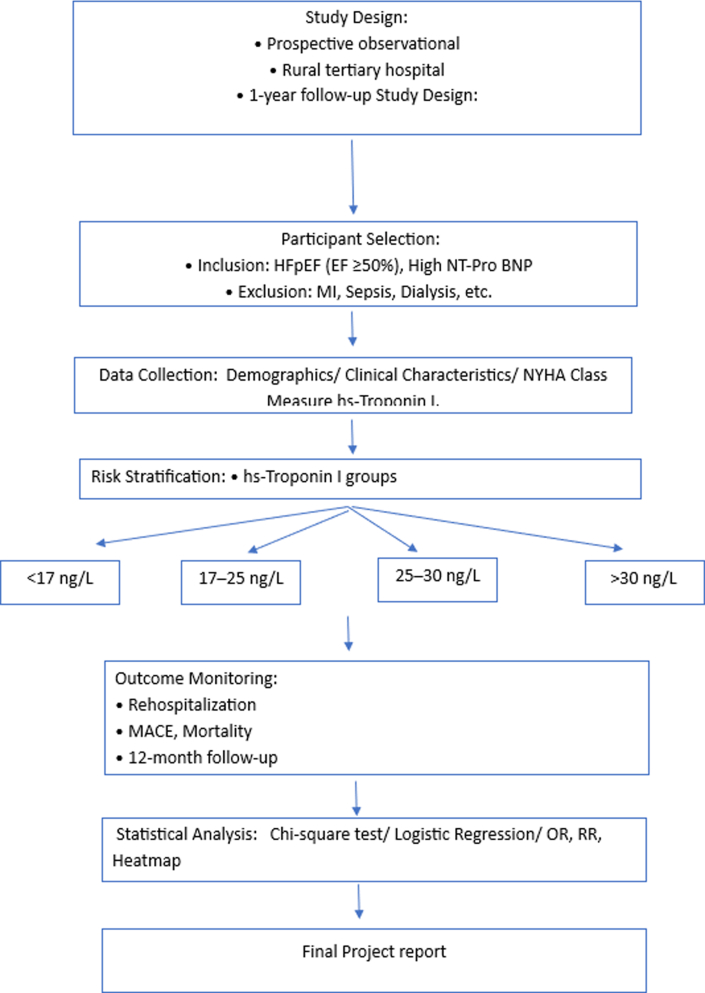
Summary of Study Design and Methodology HFpEF = heart failure with preserved ejection fraction; MACE = major adverse cardiovascular event; MI = myocardial infarction; NT-proBNP = N-terminal pro–B-type natriuretic peptide; OR = odds ratio; RR = risk ratio.

Heart failure with preserved ejection fraction (HFpEF) accounts for nearly half of all heart failure cases worldwide and is associated with substantial morbidity and mortality.^1^ Prognostic markers are essential for early risk stratification to guide management and improve outcomes. High-sensitivity cardiac troponin 1 (hs-cTn1), a biomarker of myocardial injury, has emerged as a valuable prognostic tool in HFpEF patients in well-resourced settings.^2^ However, there are limited data on its utility in low-income rural populations, where health care resources are scarce and the burden of heart failure is increasing.^3^

**Take-Home Messages**

• Elevated hs-cTn1 levels are strongly associated with increased risks of rehospitalization, major adverse cardiovascular events, and mortality in heart failure patients.
• Given its affordability and accessibility, hs-cTn1 testing can emerge as a practical tool for early risk stratification in resource-limited settings.

Patients with HFpEF have smaller left ventricular cavities and thicker walls, resulting in lower end-diastolic wall stress compared with patients with heart failure with reduced ejection fraction. This leads to reduced release of B-type natriuretic peptide, limiting its usefulness as a prognostic marker in HFpEF.^4^ Thus, there is a need to study other cost-effective biomarkers in this population.

This study aims to evaluate the potential role of hs-cTn1 as a cost-effective prognostic biomarker in HFpEF patients admitted for the first time in rural India.

## Aims and Objectives

The primary objective of this study was to assess the association of admission serum hs-cTn1 levels with heart failure severity (NYHA functional class), acute complications during hospitalization (eg, pulmonary edema, arrhythmias, hypotension, oliguria), length of hospital stay, and in-hospital mortality. The secondary objective of this study was to evaluate the relationship between hs-cTn1 level at discharge and 12-month follow-up outcomes, including rehospitalization for heart failure or major adverse cardiovascular event (MACE).

## Materials and Methods

This prospective observational study was conducted at the Akash Institute of Medical Sciences. After ethical clearance and informed consent, the study included 94 patients diagnosed with HFpEF who were evaluated for 12 months. Serum hs-cTn1 levels were measured on admission using chemiluminescent immunoassay with the Access 2 analyzer (Beckman Coulter) (sensitivity: ±2.3 ng/L, normal: <17 ng/L). Detailed clinical history, cardiovascular examination, and echocardiography assessing left ventricular systolic function were performed at admission and discharge. Heart failure severity was classified by NYHA functional class and was treated per guidelines. Follow-up occurred at 1 week, 1 month, and every 3 months thereafter to monitor rehospitalization and MACE, with telephone follow-up for those unable to attend. We excluded patients with acute coronary syndrome, those undergoing intervention or surgery during hospitalization, those with established or newly diagnosed significant coronary artery disease or chronic kidney disease requiring hemodialysis, and those with lack of consent.

## Results

The 94 study patients in this rural cohort had a mean age of 64.9 ± 10.2 years and a male predominance (61.7%). The mean body mass index was 25.7 ± 4.3 kg/m^2^. Most patients were nonsmokers (70.2%), with 16.0% being ex-smokers and 13.8% current smokers. Based on NYHA classification, 54.3% were in class II, followed by class I: 27 (28.7%); class II: 26 (27.7%); class III: 26 (27.7%); class IV: 15 (16.0%). Diabetes mellitus was highly prevalent, affecting 86.0% of the cohort. Other baseline patient characteristics, including medications, blood pressure, heart rate, and hemoglobin and creatinine values, are shown in Table 1.

**Table 1:** Baseline Characteristics of the Study Population

	Value		Value
Age, y, mean ± SD	64.9 ± 10.2	Diabetes mellitus, n (%)	81 (86.0%)
Sex, n (%)	Male: 58 (61.7%); female: 36 (38.3%)	Systolic BP, mm Hg, mean ± SD	141 ± 15
BMI, kg/m^2^, mean ± SD	25.7 ± 4.3	Diastolic BP, mm Hg, mean ± SD	88 ± 10
Smoking status, n (%)	Nonsmoker: 66 (70.2%); ex-smoker: 15 (16.0%); current smoker: 13 (13.8%)	Heart rate, beats/min, mean ± SD	75 ± 12
NYHA functional class, n (%)	Class I: 27 (28.7%); class II: 26 (27.7%); class III: 26 (27.7%); class IV: 15 (16.0%)	Creatinine, mg/dL, mean ± SD	1.2 ± 0.3
Medications at admission, n (%)	Beta-blockers: 80 (85.1%); ACE inhibitors: 42 (44.7%); loop diuretics: 85 (90.4%)	Hemoglobin, g/dL, mean ± SD	11.5 ± 1.8
NT-proBNP, pg/mL, mean ± SD	7,412 ± 5,287	hs-cTn1, ng/L, mean ± SD	27.2 ± 15.6

ACE = angiotensin-converting enzyme; BMI = body mass index; BP = blood pressure; NT-proBNP = N-terminal pro–B-type natriuretic peptide; hs-cTn1 = high-sensitivity cardiac troponin 1.

**Figure 1:**
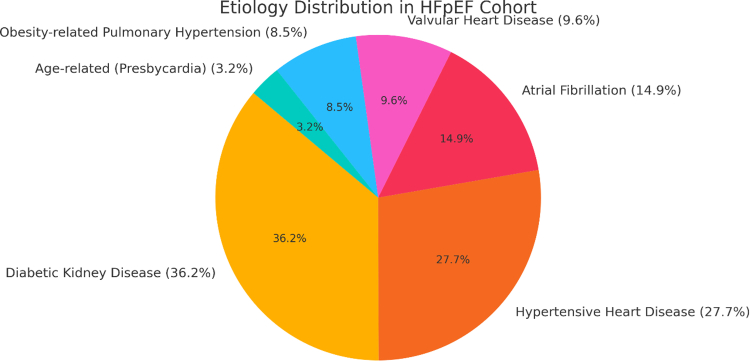
Distribution of Heart Failure Etiology in the Study Population HFpEF = heart failure with preserved ejection fraction.

Among the 94 patients, diabetic kidney disease emerged as the most prevalent etiology of HFpEF, accounting for 36.2% of cases. hypertensive heart disease followed closely, contributing to 27.7% of cases and reflecting the high burden of uncontrolled hypertension in this population. Atrial fibrillation was identified in 14.9% of patients, and valvular heart disease, primarily mitral regurgitation, accounted for 9.6%. Obesity-related pulmonary hypertension and age-related cardiac changes (presbycardia) were observed in 8.5% and 3.2%, respectively (Figure 1).

Patients with NYHA functional class III (n = 26), class IV (n = 15), and a subset of class II (n = 15) were hospitalized primarily for heart failure. These individuals presented with classic symptoms such as dyspnea, pedal edema, pulmonary edema, fluid overload, and markedly elevated N-terminal pro–B-type natriuretic peptide levels. The remaining 38 patients (40.4%) were admitted for noncardiac acute conditions during which HFpEF was diagnosed incidentally. The most frequent causes of hospitalization in this group included acute exacerbation of chronic obstructive pulmonary disease (n = 10), hypertensive emergency (n = 7), anemia requiring transfusion (n = 6), and sepsis due to systemic infections (n = 6). Less common causes were acute renal failure (n = 3), obstructive sleep apnea with pulmonary hypertension and CO_2_ retention (n = 2), thyrotoxic crisis (n = 1), mitral regurgitation with infective endocarditis (n = 1), HIV with pneumocystis jirovecii pneumonia (n = 1), and lupus nephritis (n = 1).

### Primary outcomes

A significant association was observed between hs-cTn1 level and NYHA functional class (chi-square = 16.19, *P* = 0.05). Patients with hs-cTn1 level >30 ng/L were predominantly in NYHA functional classes III and IV, whereas those with lower levels (<17 ng/L) were mostly in NYHA functional class I (Table 2).

**Table 2:** High-Sensitivity Troponin 1 Level and NYHA Functional Class

hs-cTn1 Level, ng/L	n (%)	NYHA Functional Class, n			
		Class I	Class II	Class III	Class IV
>30	11 (12%)	0	1	3	7
25-30	26 (28%)	0	5	15	6
17-25	20 (21%)	2	10	6	2
<17	37 (39%)	25	10	2	0
Total	94 (100%)	27	26	26	15

hs-cTn1 = high-sensitivity cardiac troponin 1.

Table 3 highlights the relationship between hs-cTn1 level and the clinical characteristics of the study patients. Results indicated that patients with higher hs-cTn1 level (>30 ng/L) also had a greater incidence of pulmonary edema (60.0%), atrial fibrillation (59.0%), hypotension requiring inotropes (40.0%), cardiogenic shock (36.0%), and renal dysfunction (70.0%). These patients also had longer hospital stays, with 63.6% staying >7 days. In contrast, patients with hs-cTn1 level <17 ng/L had fewer complications and shorter hospitalizations, emphasizing the prognostic value of hs-cTn1 in predicting clinical severity and outcomes.

**Table 3:** High-Sensitivity Troponin 1 Level and Clinical Characteristics

Hs-cTn1 Level, ng/L	Pulmonary Edema, %	Atrial Fibrillation, %	Mobitz Type II Heart Block, n	Hypotension Requiring Inotropes, %	Cardiogenic Shock, %	Renal Dysfunction (eGFR <60), %	Discharged ≤3 Days, %	Hospital Stay >7 Days, %
>30 (n = 11)	60.0	59.0	0	40.0	36.0	70.0	18.0	63.6
25-30 (n = 26)	54.0	45.0	0	45.0	28.0	58.0	23.0	23.1
17-25 (n = 20)	36.0	28.0	1	26.0	18.0	42.0	40.0	15.0
<17 (n = 37)	16.0	12.0	0	22.0	10.0	24.0	68.0	5.4
*P* Value	0.002	0.006	0.012	0.024	0.001	0.001	<0.001	<0.001

eGFR = estimated glomerular filtration rate; hs-cTn1 = high-sensitivity cardiac troponin 1.

### Secondary outcomes

**Figure 2:**
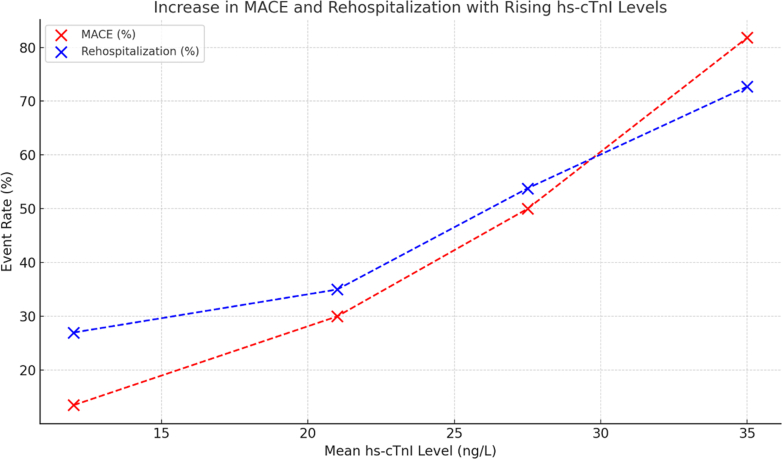
Relationship of MACE and Rehospitalization With High-Sensitivity Cardiac Troponin 1 Level hs-cTn1 = high-sensitivity cardiac troponin 1; MACE = major adverse cardiovascular event.

Table 4 demonstrates the relationship between elevated hs-cTn1 levels and adverse clinical outcomes in HFpEF patients. Those with hs-cTn1 level >30 ng/L had markedly higher rates of rehospitalization (72.7%), MACE (81.8%), and mortality (36.4%) versus those with lower levels. In contrast, patients with hs-cTn1 level <17 ng/L had the lowest event rates (Figure 2). As demonstrated in Table 5, patients with hs-cTn1 level >30 ng/L had 28.8 times higher odds of MACE and 13.45 times higher risk of mortality versus patients with hs-cTn1 level <17 ng/L.

**Table 4:** High-Sensitivity Troponin 1 Level and Adverse Outcomes After HFpEF

hs-cTn1 Level, ng/L	n	Rehospitalization for HF, n (%)	MACE, n (%)	Mortality, n (%)
>30	11	8 (72.7%)	9 (81.8%)	4 (36.4%)
25-30	26	14 (53.8%)	13 (50.0%)	2 (7.7%)
17-25	20	7 (35.0%)	6 (30.0%)	1 (5.0%)
<17	37	10 (27.0%)	5 (13.5%)	1 (2.7%)
Total	94	39 (41.5%)	33 (35.1%)	8 (8.5%)
*P* value		0.004	0.002	0.023

HF = heart failure; hs-cTn1 = high-sensitivity cardiac troponin 1; MACE = major adverse cardiovascular event.

**Table 5:** Odds Ratios and Risk Ratios for Outcomes Based on High-Sensitivity Troponin 1 Level

Outcome	Odds Ratio (95% CI)	Risk Ratio (95% CI)
MACE	28.8 (4.77-174.03)	6.05 (2.56-14.33)
Rehospitalization	7.2 (1.59-32.67)	2.69 (1.42-5.11)
Mortality	20.57 (1.99-212.72)	13.45 (1.67-108.28)

MACE = major adverse cardiovascular event.

### Binomial logistic regression analysis: hs-cTn1 and adverse outcomes

**Figure 3:**
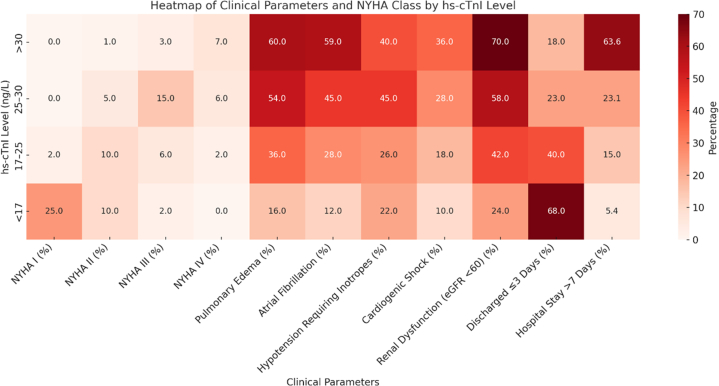
Heat Map Summarizing Study Findings eGFR = estimated glomerular filtration rate; hs-cTn1 = high-sensitivity cardiac troponin 1.

In a grouped logistic regression model, rising hs-cTn1 levels were significantly associated with increased odds of MACE (odds ratio [OR]: 1.14 per 1 ng/L increase; 95% confidence interval [CI]: 1.07-1.22; *P* < 0.001), suggesting a clear dose-response relationship between myocardial injury severity and adverse cardiovascular events in HFpEF. Elevated hs-cTn1 level also independently predicted rehospitalization (OR: 1.13; 95% CI: 1.04-1.23; *P* = 0.005) and in-hospital mortality (OR: 1.18; 95% CI: 1.04-1.35; *P* = 0.011), further underscoring its prognostic significance. Figure 3 displays a heat map summarizing the findings of the study.

## Discussion

The present study underscores the prognostic significance of hs-cTn1 levels in patients with heart failure, demonstrating an association between elevated hs-cTn1 concentrations and adverse clinical outcomes, including rehospitalization, MACE, and mortality.

Our findings align with previous research highlighting the predictive value of hs-cTn1 in patients with heart failure. A study by Kavsak et al^5^ demonstrated that elevated hs-cTn1 levels are associated with increased risk of heart failure hospitalization and mortality, emphasizing the its utility as a prognostic biomarker in both acute and chronic heart failure settings. Similarly, a meta-analysis by Noppakun et al^6^ found that higher hs-cTn1 levels correlated with increased risk of long-term mortality and MACE in patients undergoing hemodialysis, suggesting that hs-cTn1 is a valuable marker for cardiovascular risk stratification in diverse patient populations. Furthermore, our observation of a dose-response relationship between hs-cTn1 levels and adverse outcomes is consistent with the findings of Park et al,^7^ who reported that elevated hs-cTn1 concentrations are independently associated with increased risk of all-cause mortality and MACE in a community-based cohort.

## Limitations

The observational nature of the study along with the relatively low sample size of the highest hs-cTn1 group (n = 11) may limit the statistical power and generalizability of the findings. The reliance on a single hs-cTn1 measurement upon admission precludes the assessment of dynamic changes in troponin levels, which could have provided more nuanced prognostic information.

## Conclusions

The study findings highlight the prognostic value of hs-cTn1 level in predicting various complications and outcomes in patients during hospitalization for heart failure. Such stratification can aid in risk assessment and management planning for patients with elevated troponin levels.

## Funding Support and Author Disclosures

The high-sensitivity troponin 1 estimation was performed free of cost by the Department of Biochemistry, Akash Institute of Medical Sciences and Sakra World Hospital, Bengaluru, India. The authors have reported that they have no relationships relevant to the contents of this paper to disclose
